# Exploring behaviors of stochastic differential equation models of biological systems using change of measures

**DOI:** 10.1186/1471-2105-13-S5-S8

**Published:** 2012-04-12

**Authors:** Sumit Kumar Jha, Christopher James Langmead

**Affiliations:** 1 Electrical Engineering and Computer Science Department, University of Central Florida, Orlando FL 32816 USA; 2 Computer Science Department, Carnegie Mellon University, Pittsburgh PA 15213 USA; 3 Lane Center for Computational Biology, Carnegie Mellon University, Pittsburgh PA 15213 USA

## Abstract

Stochastic Differential Equations (SDE) are often used to model the stochastic dynamics of biological systems. Unfortunately, rare but biologically interesting behaviors (e.g., oncogenesis) can be difficult to observe in stochastic models. Consequently, the analysis of behaviors of SDE models using numerical simulations can be challenging. We introduce a method for solving the following problem: given a SDE model and a high-level behavioral specification about the dynamics of the model, algorithmically decide whether the model satisfies the specification. While there are a number of techniques for addressing this problem for discrete-state stochastic models, the analysis of SDE and other continuous-state models has received less attention. Our proposed solution uses a combination of Bayesian sequential hypothesis testing, *non*-identically distributed samples, and Girsanov's theorem for change of measures to examine rare behaviors. We use our algorithm to analyze two SDE models of tumor dynamics. Our use of non-identically distributed samples sampling contributes to the state of the art in statistical verification and model checking of stochastic models by providing an effective means for exposing rare events in SDEs, while retaining the ability to compute bounds on the probability that those events occur.

## Background

The dynamics of biological systems are largely driven by stochastic processes and subject to random external perturbations. The consequences of such random processes are often investigated through the development and analysis of stochastic models (e.g., [1-4]). Unfortunately, the validation and analysis of stochastic models can be very challenging [5,6], especially when the model is intended to investigate rare, but biologically significant behaviors (e.g., oncogenesis). The goal of this paper is to introduce an algorithm for examining such rare behaviors in Stochastic Differential Equation (SDE) models. The algorithm takes as input the SDE model, , and a high-level description of a dynamical behavior, *ϕ *(e.g., a formula in temporal logic). It then computes a statistically rigorous bound on the probability that the given model exhibits the stated behavior using a combination of biased sampling and Bayesian Statistical Model Checking [7,8].

**Figure 1:**
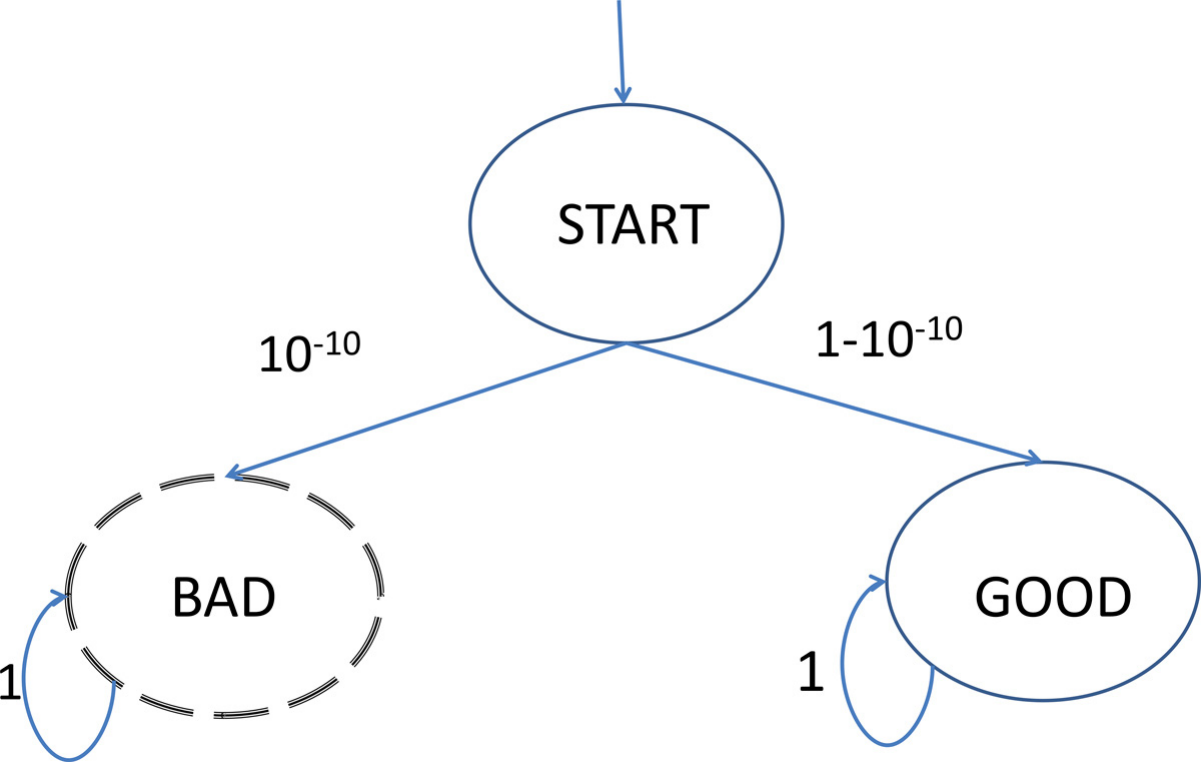
**Observing rare behaviors in i.i.d. sampling is challenging**. A toy model with a one low-probability state. An unbiased sampling algorithm may require billions of samples in order to observe the 'bad' state. Statistical algorithms based on *i.i.d*. algorithms are not suitable for analyzing such models with rare interesting behaviors.

Existing methods for validating and analyzing stochastic models often require extensive Monte Carlo sampling of independent trajectories to verify that the model is consistent with known data, and to characterize the model's expected behavior under various initial conditions. Sampling strategies are either unbiased or biased. Unbiased sampling strategies draw trajectories according to the probability distribution implied by the model, and are thus not well-suited to investigating rare behaviors. For example, if the actual probability that the model will exhibit a given behavior is 10^-10^, then the expected number of samples need to see such behaviors is about 10^10 ^(See Figure 1). Biased sampling strategies, in contrast, can be used to increase the probability of observing rare events, at the expense of distorting the underlying probability distribution/measure. If the change of measure is well-characterized (as in importance sampling), these distortions can be corrected when computing properties of the distribution.

Our method uses a combination of biased sampling and sequential hypothesis testing [9,10] to estimate the probability that the model satisfies the property. Briefly, the algorithm randomly perturbs the computational model prior to generating each sample in order to expose rare but interesting behaviors. These perturbations cause a change of measure. We note that in the context of SDEs, a change of measure is itself a stochastic process and so importance sampling, which assumes that the change of measure with respect to the biased distribution is known, cannot be used. Our technique does not require us to know the exact magnitudes of the changes of measure, nor the Radon-Nikodym derivatives [11]. Instead, it ensures that that the geometric average of these derivatives is bounded. This is a much weaker assumption than is required by importance sampling, but we will show that it is sufficient for the purposes of obtaining bounds via sequential hypothesis testing.

### Related work

Our method performs statistical model checking using hypothesis testing [12,13], which has been used previously to analyze in a variety of domains (e.g., [14-19,19]), including computational biology (e.g., [7,8,20-22]). There are two key differences between the method in this paper and existing methods. First, existing methods generate independently and identically distributed (*i.i.d*.) samples. Our method, in contrast, generates independent but *non*-identically distributed (non-*i.i.d*.) samples. It does so to expose rare behaviors. Second, whereas most existing methods for statistical model checking use classical statistics, our method employs Bayesian statistics [23,24]. We have previously shown that Bayesian statistics confers advantages in the context of statistical verification in terms of efficiency and flexibility [7,8,19,25].

## Methods

Our method draws on concepts from several different fields. We begin by briefly surveying the semantics of stochastic differential equations, a language for formally specifying dynamic behaviors, Girsanov's theorem on change of measures, and results on consistency and concentration of Bayesian posteriors.

### Stochastic differential equation models

A stochastic differential equation (SDE) [26,27] is a differential equation in which some of the terms evolve according to Brownian Motion [28]. A typical SDE is of the following form:

$$dX=b\left(t,\;X_{t}\right)dt+v\left(t,\;X_{t}\right)dW_{t}$$

where *X *is a system variable, *b *is a Riemann integrable function, *v *is an Itō integrable function, and *W *is Brownian Motion. The Brownian Motion *W *is a continuous-time stochastic process satisfying the following three conditions:

1. *W*_0 _= 0

2. *W_t _*is continuous (*almost surely*).

3. *W_t _*has independent *normally distributed *increments:

• *W_t _*- *W_s _*and *W*_*t*' _- *W*_*s*' _are independent if 0 ≤ *s *<*t *<*s*' <*t*'.

• $W_{t}-W_{s}\sim N\left(0,\;t-s\right)$, where N(0,t-s) denotes the normal distribution with mean 0 and variance *t *- *s*. Note that the symbol ~ is used to indicate "*is distributed as*".

Consider the time between 0 and *t *as divided into *m *discrete steps 0, *t*_1_, *t*_2 _... *t_m _*= *t*. The solution to a stochastic differential equation is the limit of the following discrete difference equation, as *m *goes to infinity:

$$\begin{array}{llll} X_{t_{k+1}}-X_{t_{k}} & =b\left(t_{k},\;X_{t_{k}}\right)\;\left(t_{k+1}-t_{k}\right)\; & & \;\\ & \;\;+v\left(t_{k},\;X_{t_{k}}\right)\left(W_{t_{k+1}}-W_{t_{k}}\right)\; & & \;\\ & \; & \end{array}$$

In what follows,  will refer to a system of stochastic differential equations. We note that a system of stochastic differential equations comes equipped with an inherent probability space and a natural probability measure *μ*. Our algorithm repeatedly and randomly perturbs the probability measure of the Brownian motion in the model  which, in turn, changes the underlying measure in an effort to expose rare behaviors. These changes can be characterized using Girsanov's Theorem.

#### Girsanov's theorem for perturbing stochastic differential equation models

Given a process {*θ_t _*| 0 ≤ *t *≤ *T*} satisfying the Novikov condition [29], such as an SDE, the following exponential martingale *Z_t _*defines the change from measure *P *to new measure $\hat{P}$:

$$Z_{t}=exp\left(-\int_{0}^{t}\theta_{u}dW_{u}-\int_{0}^{t}\theta_{u}^{2}du/2\right)$$

Here, *Z_t _*is the Radon-Nikodym derivative of $\hat{P}$ with respect to *P *for *t *<*T*. The Brownian motion ${Ŵ}_{t}$ under $\hat{P}$ is given by: ${Ŵ}_{t}=W_{t}+\int_{0}^{t}\theta_{u}du$. The non-stochastic component of the stochastic differential equation is not affected by change of measures. Thus, a change of measure for SDEs is a stochastic process (unlike importance sampling for explicit probability distributions).

### Specifying dynamic behaviors

Next, we define a formalism for encoding *high-level behavioral specification *that our algorithm will test against .

***Definition ***1 (Adapted Finitely Monitorable). Let *σ *be a finite-length trace from the stochastic differential equation . A specification *ϕ *is said to be *adapted finitely monitorable *(AFM) if it is possible to decide whether *σ *satisfies *ϕ*, denoted *ϕ *⊨ *ϕ*.

Certain AFM specifications can be expressed as formulas in *Bounded Linear Temporal Logic *(BLTL) [30-32]. Informally, BLTL formulas can capture the ordering of events.

***Definition ***2 (Probabilistic Adapted Finitely Monitorable). A specification *ϕ *is said to be *probabilistic adapted finitely monitorable *(PAFM) if it is possible to (deterministically or probabilistically) decide whether  satisfies *ϕ *with probability at least *θ*, denoted $M⊨P_{\geq \theta }\left(\phi \right)$.

Some common examples of PAFM specifications include Probabilistic Bounded Linear Temporal Logic (PBLTL) (e.g., see [19]) and Continuous Specification Logic. Note that temporal logic is only one means for constructing specifications; other formalisms can also be used, like Statecharts [33,34].

#### Semantics of bounded linear temporal logic (BLTL)

We define the semantics of BLTL with respect to the paths of . Let *σ *= (*s*_0_, Δ_0_), (*s*_1_, Δ_1_),... be a sampled execution of the model along states *s*_0_, *s*_1_,... with durations Δ_0_, Δ_1_, *.. *. ∈ ℝ. We denote the path starting at state *i *by *σ^i ^*(in particular, *σ*^0 ^denotes the original execution *σ*). The value of the state variable *x *in *σ *at the state *i *is denoted by *V *(*σ*, *i*, *x*). The semantics of BLTL is defined as follows:

1. *σ^k ^*⊨ *x *~ *v *if and only if *V*(*σ*, *k*, *x*) ~ *v*, where *v *∈ ℝ and ~ ∈ {>, <, =}.

2. *σ^k ^*⊨ *ϕ*_1 _∨*ϕ*_2 _if and only if *σ^k ^*⊨ *ϕ*_1 _or *σ^k ^*⊨ *ϕ*_2_.

3. *σ^k ^*⊨ *ϕ*_1 _∧ *ϕ*_2 _if and only if *σ^k ^*⊨ *ϕ*_1 _and *σ^k ^*⊨ *ϕ*_2_;

4. *σ^k ^*⊨ ¬*ϕ*_1 _if and only if *σ^k ^*⊨ *ϕ*_1 _does not hold.

5. *σ*^*k *^⊨ *ϕ*_1_**U**^*t*^*ϕ*_2 _if and only if there exists *i *∈ ℕ such that: (a) 0 ≤ *Σ*_0 ≤ *l *<*i*_Δ_*k*+*l*_≤ *t*; (b) *σ*^*k*+*i*^⊨ *ϕ*_2_; and (c) for each 0 ≤ *j < i*, *σ*^*k*+*j*^⊨ *ϕ*_1_;

### Statistical model validation

Our algorithm performs statistical model checking using Bayesian sequential hypothesis testing [23] on non-*i.i.d*. samples. The statistical model checking problem is to decide whether a model  satisfies a probabilistic adapted finitely monitorable formula *ϕ *with probability at least *θ*. That is, whether $M⊨P_{\geq \theta }\left(\phi \right)$ where *θ *∈ (0, 1).

#### Sequential hypothesis testing

Let *ρ *be the (unknown but fixed) probability of the model satisfying *ϕ*. We can now re-state the statistical model checking problem as deciding between the two *composite *hypotheses: *H*_0 _: *ρ *≽ *θ *and *H*_1 _: *ρ < θ*. Here, the *null hypothesis H*_0 _indicates that  satisfies the *AFM *formula *ϕ *with probability at least *θ*, while the *alternate hypothesis H*_1 _denotes that  satisfies the *AFM *formula *ϕ *with probability less than *θ*.

***Definition ***3 (Type I and II errors). A Type I error is an error where the hypothesis test asserts that the null hypothesis *H*_0 _is false, when in fact *H*_0 _is true. Conversely, a Type II error is an error where the hypothesis test asserts that the null hypothesis *H*_0 _is true, when in fact *H*_0 _is false.

The basic idea behind *any *statistical model checking algorithm based on sequential hypothesis testing is to iteratively sample traces from the stochastic process. Each trace is then evaluated with a trace verifier [32], which determines whether the trace satisfies the specification i.e. *σ_i _*⊨ *ϕ*. This is always feasible because the specifications used are adapted and finitely monitorable. Two accumulators keep track of the total number of traces sampled, and the number of satisfying traces, respectively. The procedure continues until there is enough information to reject either *H*_0 _or *H*_1_.

#### Bayesian sequential hypothesis testing

Recall that for any finite trace *σ_i _*of the system and an Adapted Finitely Monitorable (AFM) formula *ϕ*, we can decide whether *σ_i _*satisfies *ϕ*. Therefore, we can define a random variable *X_i _*denoting the outcome of *σ_i _*⊨ *ϕ*. Thus, *X_i _*is a *Bernoulli *random variable with probability mass function $f\left(x_{i}|\rho \right)=\rho^{x_{i}}{\left(1-\rho \right)}^{1-x_{i}}$, where *x_i _*= 1 if and only if *σ_i _*⊨*_q _ϕ*, otherwise *x_i _*= 0.

Bayesian statistics requires that *prior probability *distributions be specified for the unknown quantity, which is *ρ *in our case. Thus we will model the unknown quantity as a random variable *u *with prior density *g*(*u*). The prior probability distribution is usually based on our previous experiences and beliefs about the system. Non-informative or objective prior probability distribution [35] can be used when nothing is known about the probability of the system satisfying the *AFM *formula.

Suppose we have a sequence of *independent *random variables *X*_1_,..., *X_n _*defined as above, and let *d *= (*x*_1_,..., *x_n_*) denote a sample of those variables.

***Definition ***4. The Bayes factor  of sample *d *and hypotheses *H*_0 _and *H*_1 _is $B=\frac{P\left(d|H_{0}\right)}{P\left(d|H_{1}\right)}$.

The Bayes factor may be used as a measure of relative confidence in *H*_0 _vs. *H*_1_, as proposed by Jeffreys [36]. The Bayes factor is the ratio of two likelihoods:

(1) $$B=\frac{\int_{\theta }^{1}f\left(x_{1}|u\right)\cdots f\left(x_{n}|u\right)\cdot g\left(u\right)du}{\int_{0}^{\theta }f\left(x_{1}|u\right)\cdots f\left(x_{n}|u\right)\cdot g\left(u\right)du}.$$

We note that the Bayes factor depends on both the data *d *and on the prior *g*, so it may be considered a measure of confidence in *H*_0 _vs. *H*_1 _provided by the data *x*_1_,..., *x_n_*, and "weighted" by the prior *g*.

#### Non-i.i.d. Bayesian sequential hypothesis testing

Traditional methods for hypothesis testing, including those outlined in the previous two subsection*s, assume that the samples are drawn *i.i.d*.. In this section* we show that non-*i.i.d*. samples can also be used, provided that certain conditions hold. In particular, if one can bound the change in measure associated with the non-identical sampling, one can can also bound the Type I and Type II errors under a change of measure. Our algorithm bounds the change of measure, and thus the error.

We begin by reviewing some fundamental concepts from Bayesian statistics including KL divergence, KL support, affinity, and *δ*-separation, and then restate an important result on the concentration of Bayesian posteriors [35,37].

***Definition ***5 (Kullback-Leibler (KL) Divergence). Given a parameterized family of probability distributions {*f_θ_*}, the Kullback-Leibler (*KL*) divergence *K*(*θ*_0_, *θ*) between the distributions corresponding to two parameters *θ *and *θ*_0 _is: $K\;\left(\theta_{0},\;\theta \right)=E_{\theta_{0}}\left[f_{\theta }/f_{\theta_{{}_{0}}}\right]$. Note that $E_{\theta_{0}}$ is the expectation computed under the probability measure $f_{\theta_{0}}$.

***Definition ***6 (KL Neighborhood). Given a parameterized family of probability distributions {*f_θ_*}, the KL neighborhood *K_ε _*(*θ*_0_) of a parameter value *θ*_0 _is given by the set {*θ *: *K *(*θ*_0_, *θ*) *< ε*}.

***Definition ***7 (KL Support). A point *θ*_0 _is said to be in the KL support of a prior Π if and only if for all *ε >*0, Π (*K_ε _*(*θ*_0_)) *>*0.

***Definition ***8 (Affinity). The affinity Aff(*f*, *g*) between any two densities is defined as $\text{A}\text{f}\text{f}\left(f,\;g\right)=\int \sqrt{fg}d\mu$.

***Definition ***9 (Strong *δ*-Separation). Let *A *⊂ [0, 1] and *δ >*0. The set *A *and the point *θ*_0 _are said to be strongly *δ*-separated if and only if for any proper probability distribution *v *on *A*, $\text{A}\text{f}\text{f}\left(f_{\theta_{0}},\int_{A}f_{\theta }\left(x_{1}\right)v\left(d\theta \right)\right)<\delta$.

Given these definitions, it can be shown that the Bayesian posterior concentrates exponentially under certain technical conditions [35,37].

#### Bounding errors under a change of measure

Next, we develop the machinery needed to compute bounds on the Type-I/Type-II errors for a testing strategy based on non-*i.i.d*. samples.

A stochastic differential equation model  is naturally associated with a probability measure *μ*. Our non-*i.i.d*. sampling strategy can be thought of as the assignment of a set of probability measures *μ*_1_, *μ*_2_,... to  . Each unique sample *σ_i _*is associated with an *implied *probability measure *μ_i _*and is generated from  under *μ_i _*in an *i.i.d*. manner. Our proofs require that all the implied probability measures are *equivalent*. That is, an event is possible (resp. impossible) under a probability measure *if and only if *it is possible (resp. impossible) under the original probability measure *μ*.

We use the following result regarding change of measures. Suppose a given behavior, say *ϕ*, holds on the original model with an (unknown) probability *ρ*.

$$P\left(\rho <\theta_{0}|X_{i}\right)=\frac{\int_{0}^{\theta_{0}}p_{\mu }\left(X_{i}|u\right)\;g\left(u\right)\;du}{\int_{0}^{1}p_{\mu }\left(X_{i}|u\right)\;g\left(u\right)\;du}$$

Here, *X_i _*is a Bernoulli random variable denoting the event that *i^th ^*sample satisfies the given behavior *ϕ*. Note that the *X_i _*s must be independent of one another. Now, we can rewrite the above expression as:

$$P\;\left(\rho <\theta_{0}|X_{i}\right)=\frac{\int_{0}^{\theta_{0}}\frac{p_{\mu }\left(X_{i}|u\right)}{p_{\mu_{i}}\left(X_{i}|u\right)}p_{\mu_{i}}\left(X_{i}|u\right)g\left(u\right)\;du}{\int_{0}^{1}\frac{p_{\mu }\left(X_{i}|u\right)}{p_{\mu_{i}}\left(X_{i}|u\right)}p_{\mu_{i}}\left(X_{i}|u\right)g\left(u\right)\;du}$$

Note that the term $p_{\mu_{i}}\left(X_{i}|u\right)$denotes the probability of observing the event *X_i _*under the modified probability measure *μ_i _*if the unknown probability *ρ *were *u*. In order to ensure the independence assumption, the new probability measures *μ_i _*are chosen independently of one another. The ratio $\frac{p_{\mu_{i}}\left(X_{i}|u\right)}{p_{\mu }\left(X_{i}|u\right)}$ is the *implied Radon-Nikodym derivative *for the change of measure between two equivalent probability measures. Suppose, the testing strategy has made *n *observations *X*_1_, *X*_2_,... *X_n_*. Then,

$$P\;\left(\rho <\theta_{0}|X\right)=\frac{\int_{0}^{\theta_{0}}\overset{n}{\underset{i=1}{\prod }}\frac{p_{\mu }\left(X_{i}|u\right)}{p_{\mu_{i}}\left(X_{i}|u\right)}p_{\mu_{i}}\left(X_{i}|u\right)\;g\left(u\right)\;du}{\int_{0}^{1}\overset{n}{\underset{i=1}{\prod }}\frac{p_{\mu }\left(X_{i}|u\right)}{p_{\mu_{i}}\left(X_{i}|u\right)}p_{\mu_{i}}\left(X_{i}|u\right)\;g\left(u\right)\;du}$$

A sampling algorithm can compute $p_{\mu_{i}}\left(X_{i}|u\right)$ by drawing *independent *samples from a stochastic differential equation model under the new "modified" probability measure. We note that it is not easy to compute the change of measure $\frac{p_{\mu_{i}}\left(X_{i}|u\right)}{p_{\mu }\left(X_{i}|u\right)}$ algebraically or numerically. However, our algorithm does not need to compute this quantity explicitly. It simply establishes bounds on it.

Consider the following expression that is computable without knowing the implied Radon-Nikodym derivative or change of measure explicitly.

$$Q\;\left(\rho <\theta_{0}|X_{i}\right)=\frac{\int_{0}^{\theta_{0}}p_{\mu_{i}}\left(X_{i}|u\right)\;g\left(u\right)\;du}{\int_{0}^{1}p_{\mu_{i}}\left(X_{i}|u\right)\;g\left(u\right)\;du}$$

Now, we can rewrite the above expression as:

$$Q\;\left(\rho <\theta_{0}|X_{i}\right)=\frac{\int_{0}^{\theta_{0}}\frac{p_{\mu_{i}}\left(X_{i}|u\right)}{p_{\mu }\left(X_{i}|u\right)}\;p_{\mu }\left(X_{i}|u\right)\;g\left(u\right)\;du}{\int_{0}^{1}\frac{p_{\mu_{i}}\left(X_{i}|u\right)}{p_{\mu }\left(X_{i}|u\right)}\;p_{\mu }\left(X_{i}|u\right)\;g\left(u\right)\;du}$$

Our result will exploit the fact that we do not allow our testing or sampling procedures to have arbitrary implied Radon-Nikodym derivatives. This is reasonable as no statistical guarantees should be available for an intelligently designed but adversarial test procedure that (say) tries to avoid sampling from the given behavior. Suppose that the implied Radon-Nikodym derivative always lies between a constant *c *and another constant 1*/c*. That is, the change of measure does not distort the probabilities of observable events by more than a factor of *c*. Then, we observe that:

$$\begin{array}{ccc} Q\;\left(\rho <\theta_{0}|X_{i}\right) & \leq & \frac{\int_{0}^{\theta_{0}}cp_{\mu }\left(X_{i}|u\right)\;g\left(u\right)\;du}{\int_{0}^{1}\frac{1}{c}p_{\mu }\left(X_{i}|u\right)\;g\left(u\right)\;du}\\ & = & c^{2}P\left(\rho <\theta_{0}|X_{i}\right) \end{array}$$

Furthermore,

$$Q\;\left(\rho <\theta_{0}|X_{i}\right)\;\geq \;\frac{1}{c^{2}}P\;\left(\rho <\theta_{0}|X_{i}\right).$$

Thus, by allowing the sampling algorithm to change measures by at most *c*, we have changed the posterior probability of observing a behavior by at most *c*^2^.

**Example: **Suppose, the testing strategy has made *n *observations *X*_1_, *X*_2_,... *X_n_*. Then,

$$\begin{array}{ccc} Q\;\left(\rho <\theta_{0}|X\right) & = & \frac{\int_{0}^{\theta_{0}}\overset{n}{\underset{i=1}{\prod }}\frac{p_{\mu_{i}}\left(X_{i}|u\right)}{p_{\mu }\left(X_{i}|u\right)}\;p_{\mu }\left(X_{i}|u\right)\;g\left(u\right)\;du}{\int_{0}^{1}\overset{n}{\underset{i=1}{\prod }}\frac{p_{\mu_{i}}\left(X_{i}|u\right)}{p_{\mu }\left(X_{i}|u\right)}\;p_{\mu }\left(X_{i}|u\right)\;g\left(u\right)\;du}\\ & \leq & \frac{\int_{0}^{\theta_{0}}c^{n}\overset{n}{\underset{i=1}{\prod }}\left(p_{\mu }\left(X_{i}|u\right)\right)\;g\left(u\right)\;du}{\int_{0}^{1}\frac{1}{c^{n}}\overset{n}{\underset{i=1}{\prod }}\left(p_{\mu }\left(X_{i}|u\right)\right)\;g\left(u\right)\;du}\\ & = & c^{2n}P\;\left(\rho <\theta_{0}|X_{1},\;X_{2},\;\ldots X_{n}\right) \end{array}$$

Similarly,

$$\begin{array}{ccc} Q\;\left(\rho <\theta_{0}|X\right) & \geq & \frac{1}{c^{2n}}\frac{\int_{0}^{\theta_{0}}\overset{n}{\underset{i=1}{\prod }}\left(p_{\mu }\left(X_{i}|u\right)\right)\;g\left(u\right)\;du}{\int_{0}^{1}\overset{n}{\underset{i=1}{\prod }}\left(p_{\mu }\left(X_{i}|u\right)\right)\;g\left(u\right)\;du}\\ & = & \frac{1}{c^{2n}}P\;\left(\rho <\theta_{0}|X_{1},\;\ldots X_{n}\right) \end{array}$$

#### Termination conditions for non-i.i.d. sampling

Traditional (i.e., *i.i.d*.) Bayesian Sequential Hypothesis Testing is guaranteed to terminate. That is, only a finite number of samples are required before the test selects one of the hypotheses. We now consider the conditions under which a Bayesian Sequential Hypothesis Testing based procedure using non-*i.i.d*. samples will terminate. To do this, we first need to show that the posterior probability distribution will concentrate on a particular value as we see more an more samples from the model.

To consider the conditions under which our algorithm will terminate after observing *n *samples, note that the factor introduced due to the change of measure *c*^2*n *^can outweigh the gain made by the concentration of the probability measure *e*^-*nb*^. This is not surprising because our construction thus far does not force the test *not *to bias against a sample in an intelligent way. That is, a maliciously designed testing procedure could simply avoid the error prone regions of the design. To address this, we define the notion of a *fair *testing strategy that does not engage in such malicious sampling.

***Definition ***10. A testing strategy is *η*-**fair **(*η *≥ 1) if and only if the geometric average of the implied *Radon-Nikodym derivatives *over a number of samples is within a constant factor *η *of unity, i.e.,

$$\frac{1}{\eta }\;\leq \;\sqrt[{n}]{\overset{n}{\underset{i=1}{\prod }}\frac{p_{\mu_{i}}\left(X_{i}|u\right)}{p_{\mu }\left(X_{i}|u\right)}}\leq \eta$$

Note that a fair test strategy does *not *need to sample from the underlying distribution in an *i.i.d*. manner. However, it *must *guarantee that the probability of observing the given behavior in a large number of observations is not altered *substantially *by the non-*i.i.d*. sampling. Intuitively, we want to make sure that we bias *for *each sample as many times as we bias *against *it. Our main result shows that such a long term neutrality is sufficient to generate statistical guarantees on an otherwise non-*i.i.d*. testing procedure.

***Definition ***11. An *η*-**fair **test is said to be **eventually fair **if and only if 1 ≤ *η*^4 ^<*e^b^*, where *b *is the constant in the exponential posterior concentration theorem.

The notion of a *eventually fair *test corresponds to a testing strategy that is not malicious or adversarial, and is making an honest attempt to sample from all the events in the long run.

### Algorithm

**Figure 2:**
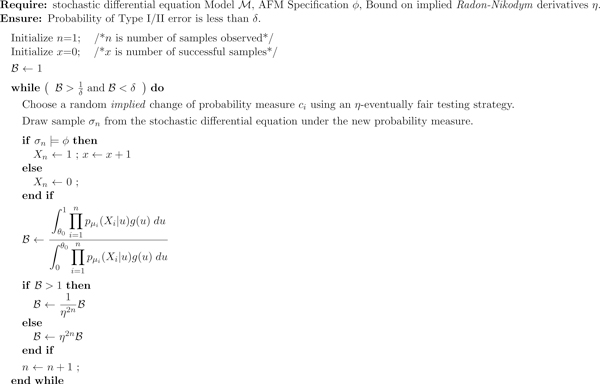
**Non-i.i.d. Statistical Verification Algorithm**. The figure illustrated the *non-i.i.d*. Bayesian model validation algorithm. The algorithm builds upon Girsanov's theorem on change of measure and Bayesian model validation.

Finally, we present our Statistical Verification algorithm (See Figure 2) in terms of a generic non-*i.i.d*. testing procedure sampling with random "implied" change of measures. Our algorithm is relatively simple and generalizes our previous Bayesian Statistical verification algorithm [8] to non-*i.i.d*. samples using change of measures. The algorithm draws non-*i.i.d*. samples from the stochastic differential equation under randomly chosen probability measures. The algorithm ensures that the implied change of measure is bounded so as to make the testing approach fair. The variable *n *denotes the number of samples obtained so far and *x *denoted the number of samples that satisfy the AFM specification *ϕ*. Based upon the samples observed, we compute the Bayes Factor under the new probability measures. We know that the Bayes Factor so computed is within a factor of the original Bayes Factor under the natural probability measure. Hence, the algorithm divides the Bayes Factor by the factor *η*^2*n *^if the Bayes Factor is larger than one. If the Bayes Factor is less than one, the algorithm multiplies the Bayes Factor by the factor *η*^2*n*^.

## Results and discussion

We applied our algorithm to two SDE models of tumor dynamics from the literature. The first model is a single dimensional stochastic differential equation for the influence of chemotherapy on cancer cells, and the second model is a pair of SDEs that describe an immunogenic tumor.

### Lefever and Garay model

Lefever and Garay [38] studied the growth of tumors under immune surveillance and chemotherapy using the following stochastic differential equation:

$$\begin{array}{llll} \frac{dx}{dt} & =r_{0}x\;\left(1-\frac{x}{K}\right)-\frac{\beta x^{2}}{1+x^{2}}+x\left(1-\frac{x}{K}\right)\;A_{0}cos\;\left(\omega t\right)\; & & \;\\ & \;\;\;+x\;\left(1-\frac{x}{K}\right)\;W_{t}\; & & \;\\ & \; & \end{array}$$

Here, *x *is the amount of tumor cells, *A*_0_*cos*(*ωt*) denotes the influence of a periodic chemotherapy treatment, *r*_0 _is the linear per capita birth rate of cancer cells, *K *is the carrying capacity of the environment, and *β *represents the influence of the immune cells. Note that *W_t _*is the standard Brownian Motion, and default model parameters were those used in [38]..

We demonstrate our algorithm on a simple property of the model. Namely, starting with a tumor consisting of a billion cells, is there at least a 1% chance that the tumor could increase to one hundred billion cells under under immune surveillance and chemotherapy. The following BLTL specification captures the behavioral specification:

$$Pr_{\geq 0.01}\left(F^{10}\left(x>10^{11}\right)\right)$$

**Figure 3:**
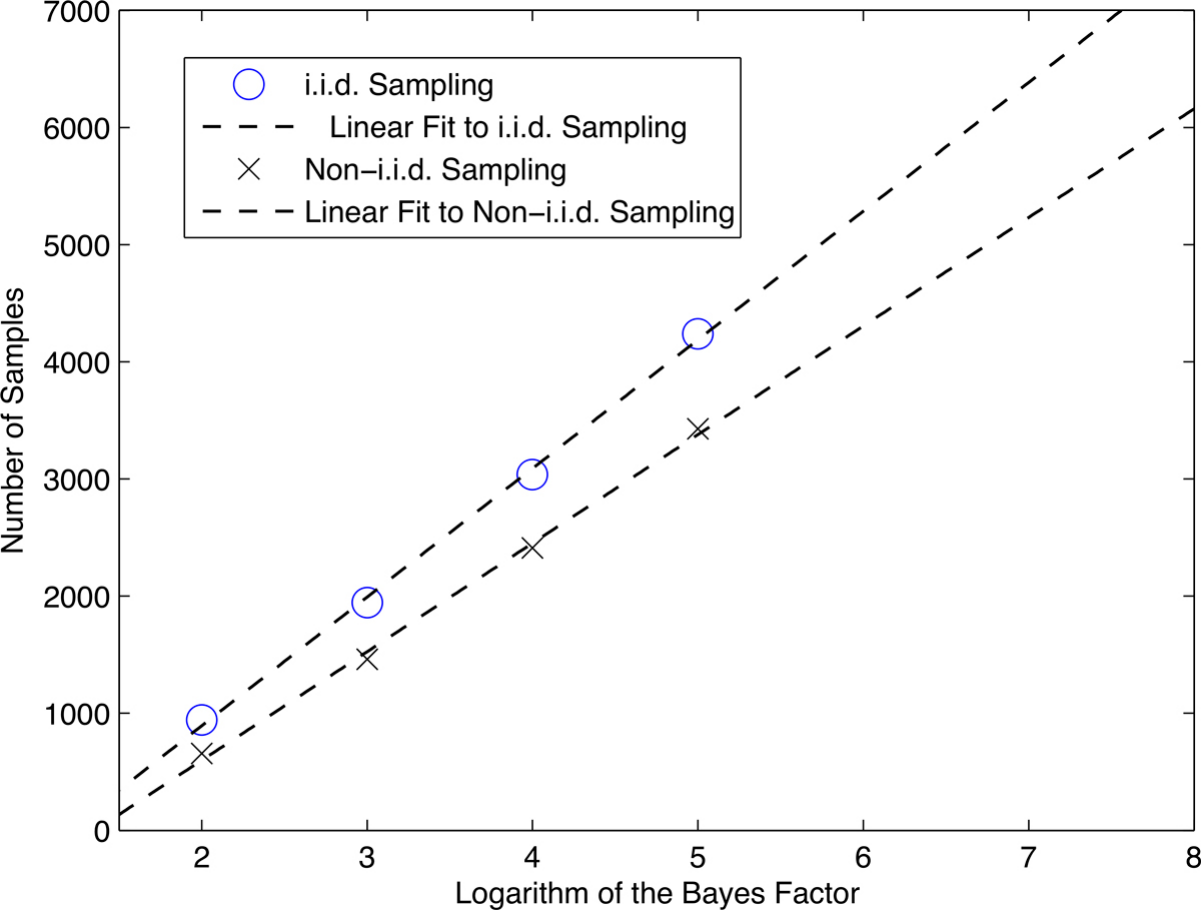
**Comparison of i.i.d. and non-i.i.d. sampling**. Non-i.i.d. vs i.i.d. sampling based verification for the Lefever and Garay model.

Figure 3 contrasts the number of samples needed to decide whether the model satisfies the property using *i.i.d*. and non-*i.i.d*.. As expected, the number of samples required increases linearly in the logarithm of the Bayes factor regardless of whether *i.i.d*. or non-*i.i.d*. sampling is used. However, non-*i.i.d*. sampling always requires fewer samples than *i.i.d*. sampling. Moreover, the difference between the number of samples increases with the Bayes factor. That is, the lines are diverging.

We note that there are circumstances when our algorithm may require *more *samples than one based on *i.i.d*. sampling. This will happen when the property being tested has relatively high probability. For example, we tested the property that the probability of eradicating the tumor is *at most *1%. The *i.i.d*. algorithm required 1374 samples to deny this possibility while the non-*i.i.d*. algorithm required 1526 samples. Thus, our algorithm is best used to examine rare behaviors.

### Nonlinear immunogenic tumor model

The second model we analyze studies immunogenic tumor growth [39,40]. Unlike the previous model, the immunogenic tumor model explicitly tracks the dynamics of the immune cells (variable *x*) in response to the tumor cells (variable *y*). The SDEs are as follows:

$$\begin{array}{ccc} dx\left(t\right) & = & \left(a_{1}-a_{2}x\;\left(t\right)+a_{3}x\;\left(t\right)y\left(t\right)\right)\;dt\\ & & +\;\left(b_{11}\left(x\left(t\right)-x_{1}\right)+b_{12}\left(y\left(t\right)-y_{1}\right)\right)\;dW_{1}\left(t\right)\\ dy\left(t\right) & = & \left(b_{1}y\left(t\right)\;\left(1-b_{2}y\left(t\right)\right)-x\left(t\right)y\left(t\right)\right)\;dt\\ & & +\;\left(b_{21}\left(x\left(t\right)-x_{1}\right)+b_{22}\left(y\left(t\right)-y_{1}\right)\right)\;dW_{2}\left(t\right) \end{array}$$

The parameters *x*_1 _an *y*_1 _denote the stochastic equilibrium point of the model. Briefly, the model assumes that the amount of noise increases with the distance to the equilibrium point.

For this model, we considered the following property: starting from 0.1 units each of tumor and immune cells, is there at least a 1% chance that the number of tumor cells could increase to 3.3 units. The property can be encoded into the following BLTL specification:

$$Pr_{\geq 0.01}\left(F^{10}\left(x>3.3\right)\right)$$

**Figure 4:**
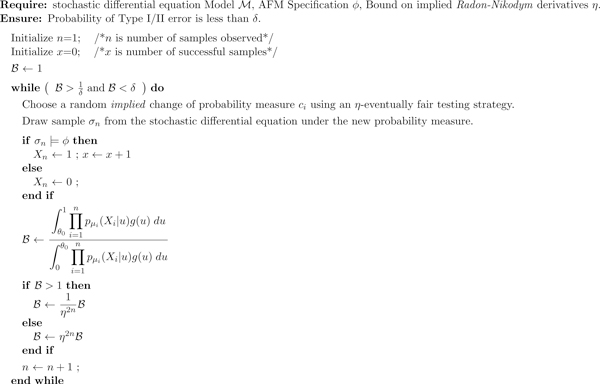
**Comparison of i.i.d. and non-i.i.d. sampling**. Non-i.i.d. vs i.i.d. Sampling based verification for the nonlinear Immunogenic tumor model.

Default model parameters were those used in [39]. Figure 4 contrasts the number of samples needed to decide whether the model satisfies the property using *i.i.d*. and non-*i.i.d*.. The same trends are observed as in the previous model. That is, our algorithm requires fewer samples than *i.i.d*. hypothesis testing, and that the difference between these methods increases with the Bayes factor.

We also considered the property that the number of tumor cells increases to 4.0 units. We evaluated whether this property is true with probability at least 0.000005 under a Bayes Factor of 100, 000. The *i.i.d*. sampling algorithm did not produce an answer even after observing 10, 000 samples. The non-*i.i.d*. model validation algorithm answered affirmatively after observing 6, 736 samples. Once again, the real impact of the proposed algorithm lies in uncovering rare behaviors and bounding their probability of occurrence.

## Discussion

Our results confirm that non-*i.i.d*. sampling reduces the number of samples required in the context of hypothesis testing -- when the property under consideration is rare. Moreover, the benefits of non-*i.i.d*. sampling increase with the rarity of the property, as confirmed by the divergence of the lines in Figures 2 and 3. We note, however, that if the property isn't rare then a non-*i.i.d*. sampling strategy will actually require a larger number of samples than an *i.i.d*. strategy. Thus, our algorithm is only appropriate for investigating rare behaviors.

## Conclusions

We have introduced the first algorithm for verifying properties of stochastic differential equations using sequential hypothesis testing. Our technique combines Bayesian statistical model checking and non-*i.i.d*. sampling and provides guarantees in terms of termination, and the number of samples needed to achieve those bounds. The method is most suitable when the behavior of interest is the exception and not the norm.

The present paper only considers SDEs with independent Brownian noise. We believe that these results can be extended to handle SDEs with certain kinds of correlated noise. Another interesting direction for future work is the extension of these method to stochastic partial differential equations, which are used to model spatially inhomogeneous processes. Such analysis methods could be used, for example, to investigate properties concerning spatial properties of tumors, the propagation of electrical waves in cardiac tissue, or more generally, to the diffusion processes observed in nature.

## Competing interests

The authors declare that they have no competing interests.

## Authors' contributions

SKJ and CJL contributed equally to all parts of the paper.
